# JAK Inhibition in Patients with Rheumatoid Arthritis: Haemodynamic Effects and Impact on Micro- and Macrovascular Function. Study Design and Rationale

**DOI:** 10.31138/mjr.33.4.471

**Published:** 2022-12-31

**Authors:** Panagiota Anyfanti, Elena Angeloudi, Eleni Pagkopoulou, Eleni Bekiari

**Affiliations:** 1 Second Medical Department, Hippokration Hospital, Aristotle University of Thessaloniki, Thessaloniki, Greece,; 2 Fourth Department of Internal Medicine, Hippokration Hospital, Aristotle University of Thessaloniki, Thessaloniki, Greece

**Keywords:** rheumatoid arthritis, Janus kinase inhibitors, blood pressure, nailfold videocapillaroscopy, arterial stiffness

## Abstract

Rheumatoid arthritis (RA) is characterised by increased rates of cardiovascular disease (CVD), which represents the leading cause of death. Patients with RA presents increased prevalence of hypertension, which substantially contributes to the increased CVD burden associated with the disease. A solid pathophysiological background supports the presence of microvascular dysfunction in RA even in the absence of established CVD, while macrovascular dysfunction in the form of large artery stiffening has been further described. Janus kinase (JAK) inhibitors constitute a novel class of disease-modifying anti-rheumatic drugs (DMARDs) for the management of rheumatoid arthritis (RA). However, the vascular effects of JAK inhibitors in RA patients remain largely understudied. More recent evidence suggests higher risk of major adverse cardiovascular events with JAK inhibition compared to treatment with a TNF inhibitor, and calls for more careful consideration of potential negative effects on the cardiovascular system. The present prospective observational cohort study aims to investigate the impact of JAK inhibitors on ambulatory blood pressure and haemodynamic profile, as well as markers of micro- and macrovasculopathy among patients with RA.

## INTRODUCTION

Rheumatoid arthritis (RA) is the most prevalent autoimmune rheumatic disorder characterised by increased rates of cardiovascular complications, which represent the leading cause of death in these patients.^1,2^ Microvascular endothelial dysfunction represents a primary mechanistic model for the development and progression of cardiovascular disease (CVD), as it precedes and accelerates large artery dysfunction, systemic atherosclerosis, and related risk factors. A solid pathophysiological background supports the presence of vascular dysfunction in RA, which is detected even in the absence of established CVDs.^3–5^ Dysregulation of autoimmunity and cumulative inflammatory load in RA leads to overexpression of adhesion molecules, pro-thrombotic, and pro-inflammatory factors, which in turn increase vascular smooth muscle cell proliferation and tone and promote the development of atherosclerosis.^6^ Indeed, patients with RA show impaired vascular biomarkers such as flow-mediated dilation (FMD) of the brachial artery.^7^ Furthermore, elevated levels of circulating biomarkers of thromboinflammation and endothelial dysfunction in patients with autoimmune inflammatory diseases suggest that an ongoing vasculodestructive process is already underway despite the absence of clinical evidence of CVD.^8^

While microvascular endothelial dysfunction is considered inherent to the disease, patients with RA present increased prevalence of traditional CVD risk factors.^9^ Among these patients, hypertension represents the most prevalent CVD risk factor and significantly aggravates cardiovascular prognosis.^10^ Despite major implications in terms of CVD development and progression, hypertension remains largely underdiagnosed and undertreated in patients with RA.^11^ Furthermore, the impact of anti-rheumatic treatment is often neglected and needs to be further taken into consideration. Antirheumatic treatment modalities exert divergent effects on the vasculature, with positive, neutral, or even negative effects which further contribute to the CVD complications associated with RA. While these effects have been well-described or are being intensively investigated for cornerstone treatments such as corticosteroids or biologics, the vascular effects of novel antirheumatic treatments, such Janus kinase (JAK) inhibitors, remain in comparison understudied.^12,13^ Ultimately, chronic inflammation, traditional CVD risk factors and, to a different extent, specific antirheumatic treatment modalities act synergistically to form a prothrombotic microenvironment and trigger the degeneration and remodelling of the vascular architecture.

## JAK INHIBITION AND CARDIOVASCULAR COMPLICATIONS

JAK inhibitors are the newest drug class of disease-modifying medication that have been approved for the treatment of RA. Their mechanism of action is based on the modification of the signal transducer and activator of transcription (STAT) constituents in cytokine signalling, which is implicated in the pathogenesis of RA. As a targeted low molecular mass drug that can pass the lipid bilayer of the cellular membrane, they offer the first long-term oral biologic option in RA.^14^ In the 2019 updated European League Against Rheumatism (EULAR) recommendations for the management of RA with synthetic and biological disease-modifying antirheumatic drugs (DMARDs), it is recommended to add any biological DMARD or JAK inhibitor on top of conventional synthetic DMARDs upon presence of poor prognostic factors (presence of autoantibodies, high disease activity, early erosions or failure of two csDMARDs). Potential safety concerns secondary to JAK inhibition include a higher risk of herpes zoster infections and venous thromboembolic events (deep vein thrombosis, pulmonary embolism).^15^ With regard to the latter, the effects of JAK inhibitors on the cardiovascular system are being thoroughly investigated and have not yet been specified in detail. A systematic review and meta-analysis of randomised controlled trials indicated no significant change in cardiovascular risk for patients with RA treated with JAK inhibitors at least in a short-term perspective.^16^ Nevertheless, the recent ORAL Surveillance trial comparing the combined tofacitinib doses with a TNF inhibitor in a cardiovascular risk–enriched population with RA showed higher risks of major adverse cardiovascular events and cancer with the JAK inhibitor than with a TNF inhibitor.^17^ No apparent mechanism of action has been proposed or proven currently for this safety signal.^18^ Despite their efficacy in the suppression of RA-related inflammation, treatment with JAK inhibitors may negatively affect CVD risk factors, such as platelet count and lipidemic profile.^19^ By contrast, data remain scarce regarding the impact of JAK inhibitors on blood pressure, especially ambulatory blood pressure, haemodynamic profile, and markers of micro- and macrovasculopathy among patients with RA. Notably, both the macrovasculature and the microvascular network in RA can be easily assessed by use of non-invasive techniques that are being widely applied in the field of CVDs, such as assessment of arterial stiffness and nailfold videocapillaroscopy (NVC), respectively.

## 24H AMBULATORY BLOOD PRESSURE

24h ambulatory blood pressure is a powerful predictor of CVD morbidity and mortality in the general population and in high CVD risk populations.^20^ Its predictive value in terms of cardiovascular health is superior to the conventional office blood pressure measurements, as stated in current hypertension guidelines.^21^ Patients with RA present increased prevalence of hypertension, which along with smoking confers the highest CVD risk of all traditional CVD risk factors in this specific group of patients.^9^ They additionally present disturbed abnormal blood pressure rhythm, with increased prevalence of nocturnal hypertension and disturbed nocturnal blood pressure drop (non-dipping).^22^ Remarkably, the newest devices for ambulatory blood pressure monitoring can simultaneously record haemodynamic parameters throughout the whole 24h period, including central (aortic) blood pressure, wave reflection, total vascular resistance, as well as markers of arterial stiffness such as augmentation index (AIx) and pulse wave velocity (PWV).

## ARTERIAL STIFFNESS AS A SURROGATE CVD RISK MARKER

Arterial stiffness is defined as the loss of arterial elasticity and compliance subsequent to mechanical and structural changes of the vascular wall. The gold-standard marker of arterial stiffness is measurement of PWV, which corresponds to the rate at which pressure waves travel downstream through the arterial wall.^23^ Other markers of arterial stiffness include the assessment of AIx from the analysis of the wave reflections, and pulse pressure calculated by subtracting diastolic blood pressure from systolic blood pressure.^24^ Data from tenths of thousands of individuals have established the prognostic value of arterial stiffness in terms of CVD risk prediction.^25^ Arterial stiffness is closely associated with traditional CVD risk factors, predominantly age and hypertension.^24^ Increased PWV is an independent predictor of CVD morbidity and mortality in the general population and high risk patients such as those with hypertension, type 2 diabetes mellitus, end-stage renal disease, and elderly individuals.^25,26^ Its predictive value not only remains significant even after adjustment for Framingham risk score,^27^ but also enhances the utility of current risk prediction models when added on top of classical CVD risk factors.^28^ Arterial stiffness in RA has been addressed in several studies, with the majority concluding that markers of arterial stiffness are increased in RA individuals. However, whether and to which extent increased arterial stiffness in RA reflects the increased prevalence of traditional CVD risk factors or the consequences of chronic inflammation remain under investigation.^29^ Furthermore, the impact of novel antirheumatic treatments including JAK inhibitors on arterial stiffness remains unclear.

## NVC AS MARKER OF MICROVASCULAR HEALTH AND DISEASE

The dermal capillary network represents an “open window” for the study of the microcirculation. NVC has been widely applied as a bedside aid in rheumatology, especially in the diagnosis and follow-up of patients with systemic sclerosis. In these patients, peripheral microvascular abnormalities detected with NVC reflect the severity of visceral organ involvement^30^ and correlate with the presence of pulmonary arterial hypertension^31^ and as markers of CVD risk, such as arterial stiffness^32,33^ and increased levels of uric acid.^34^ Capillaroscopic microvascular abnormalities are also common in patients with CVDs. Evaluation of NVC in autoimmune rheumatic diseases has been proposed as a potential tool for the prediction of microvascular heart involvement,^35^ while preliminary data have shown that capillary abnormalities correlate with CVD risk, either as an individual marker in high-risk patients^36^ or in combination with other microvascular indices in low-to-moderate risk hypertensive patients.^37^ Several patterns of capillaroscopic abnormalities have been previously described with NVC in patients with diabetes mellitus including capillary dilatation, avascular zones and tortuous capillaries,^38^ while presence of diabetes mellitus further aggravates structural and functional capillary density in patients with chronic kidney disease.^39^ Capillary rarefaction is particularly common in patients with hypertension, both as a cause and consequence of high blood pressure.^40^ Last but not least altered capillaroscopic parameters indicative of peripheral microangiopathy have been described in patients with pulmonary arterial hypertension associated with various autoimmune diseases^41^ or due to congenital heart disease,^42^ and chronic thromboembolic pulmonary hypertension.^43^ Dermal capillary alterations detected with NVC and their clinical significance are being thoroughly investigated in patients with RA and have been summarized in detail elsewhere.^44^

## AIM OF THE STUDY

The present study aims to provide further insight on the effects of treatment with JAK inhibitors on blood pressure and haemodynamic parameters, microvascular function, and subclinical macrovascular injury among patients with RA. More specifically, we aim to investigate whether treatment with JAK inhibitors in patients with RA induces alterations in:

1. 24h BP and its circadian rhythm
2. Arterial stiffness, specifically AIx and PWV which represent the most widely used markers of large artery stiffening.
3. Microangiopathy of the nailfold capillaries.
4. Patients’ haemodynamic profile throughout a whole 24h period (central aortic blood pressure, wave reflection, total vascular resistance)
5. Prothrombotic markers
6. Estimated cardiovascular risk

## METHODS

### Study design and setting

This is a prospective, observational cohort study, that will be conducted by the Fourth Internal Medicine Department at Hippokrateion General Hospital of Thessaloniki in collaboration with the Second Medical Department of the same hospital.

### Inclusion criteria

This study enrols adult patients with an established diagnosis of RA according to the American Rheumatism Association 1987 Revised Criteria,^45^ who are eligible for treatment with JAK inhibitors (tofacitinib, baricitinib or upadacitinib) for first time, irrespective of previous administration of conventional or biologic DMARDs based on international recommendations (EULAR 2020).^15^

Exclusion criteria include: age <18 years, inability to comprehend and sign the informed consent, previous exposure to JAK inhibitors, concomitant active malignancy or other disease with poor prognosis, recent major adverse cardiovascular event (myocardial infarction, unstable angina, stroke) within the past 6 months, and stage III–IV heart failure according to the New York Heart Association (NYHA) criteria.^46^

### Study overview

RA patients eligible for treatment with JAK inhibitors who will be selected on the basis of inclusion and exclusion criteria to participate in the research protocol will undergo the following procedures, before the initiation of JAK inhibitors:

1. Recording of medical history and current medical treatment
2. Anthropometric measurements, clinical examination and assessment of disease severity with calculation of DAS28 (Disease Activity Score in 28 joints)
3. Evaluation of functional ability and quality of life through specifically designed questionnaires
4. Office blood pressure recording
5. Estimation of 10-year cardiovascular risk
6. Videocapillaroscopy of the nailfold capillary network
7. Assessment of 24h blood pressure, its day- to night-time variation and haemodynamic parameters with the Mobil-O-Graph device
8. Blood sampling, centrifugation and thawing of the supernatant

All patients will be re-evaluated and the above procedures will be repeated at 3 and 6 months following the baseline visit, whilst the patients are under treatment with JAK inhibitors.

### Study procedures

#### Measurement of blood pressure, arterial stiffness, and haemodynamic parameters

24h blood pressure recording is recommended as the ideal method of BP monitoring according to current hypertension guidelines.^21^ The Mobil-O-Graph (IEM, Stolberg, Germany) device will be applied to all participants, which has been approved by Food and Drug Administration (FDA) and the European Union and has been validated according to the protocols of the European and the British Society of Hypertension^47,48^. Automatic BP readings will be performed at 20-min intervals during the day and at 30-min intervals during the night. Only ABPM reports with a minimum of 70% successful readings will be regarded as technically sufficient. Mean 24h, day- and night-time BP will be obtained, as well as the nocturnal fall in BP (%), also defined as “% dipping”. According to the dipping values, individuals will be classified as “dippers” (≥10%) and “non-dippers” (<10%).

Moreover, arterial stiffness indices (PWV, AIx) and haemodynamic parameters (central [aortic] systolic, diastolic and mean blood pressure, pulse pressure, total vascular resistance, and reflection coefficient) are simultaneously obtained at each blood pressure measurement. The mean values of the above markers will be included in the analysis.

#### Nailfold videocapillaroscopy

NVC is an established non-invasive method to assess the capillary vasculature of the digital arteries, which provides information on the structural and functional abnormalities of the capillaries. It is performed at room temperature (22 °C–23 °C) with the patient seated and resting for 15 minutes. NVC will be performed at the Fourth Internal Medicine Department at Hippokrateion Hospital using an Optilia Digital Capillaroscope. Pictures and videos of 15 seconds duration will be captured using the 200x magnification video camera from the 2nd to the 5th finger of each hand. The images will be analysed with Optipix capillaroscopy software 1.7.x on a computer. The NVC parameters to be measured are: capillary density, defined as the number of capillaries in the first row in 1mm (capillaries / mm), capillary width and length (μm), the presence of micro-bleeding, oedema, thrombi, and the presence of any abnormalities in the morphology or disorder of capillary architecture. These parameters will be determined on each nail and the average of all measurements for each patient will be calculated at the end. In addition to quantitative and qualitative parameters, a semi-quantitative score will be established to assess the severity of peripheral microangiopathy.

#### Blood tests

Routine blood tests will be performed for the evaluation of haematological and biochemical parameters (glucose, lipid levels, renal function, uric acid), erythrocyte sedimentation rate, and C-reactive protein.

In addition, blood samples will be collected, centrifuged and frozen at −80°C. Levels of the following prothrombotic markers will be simultaneously measured with ELISA (enzyme-linked immunosorbent assay) using commercially available kits: von Willebrand Factor antigen (vWF:Ag) and activity (vWF:RCo), thrombomodulin and ADAMTS13 activity.

#### Estimation of cardiovascular risk

Cardiovascular risk calculators specifically developed for RA patients have not been proved to be superior to the general population cardiovascular risk calculators, including Framingham Risk Score^49^. In our study, the relevant algorithm from the Framingham Heart Study will be used to calculate 10-year risk of general cardiovascular disease.^50^

## STUDY OUTCOMES

### Primary outcomes

To determine potential differences at 3 and 6 months following treatment with JAK inhibitors compared to baseline, in the following parameters: a) 24h BP, b) arterial stiffness, c) microangiopathy of dermal capillary network, d) prothrombotic markers.

### Secondary Outcomes

- To determine potential differences in the haemodynamic profile and the estimated 10-year cardiovascular risk of patients with RA at 3 and 6 months post-treatment with JAK inhibitors, compared to the baseline visit.
- To detect potential associations of prothrombotic markers with 24h BP, arterial stiffness and microangiopathy of the dermal capillary network.

## SAMPLE SIZE

A total of 30–50 patients will be studied.

## STATISTICAL METHODS

Data analysis will be performed using SPSS (Statistical Package for Social Sciences, SPSS Inc., Chicago, IL, USA) software, version 22. Results will be expressed as frequencies for qualitative variables, and as mean±standard deviation (m±SD) or median (interquartile range) for continuous variables. Comparisons of variables will be performed between the baseline and the follow-up visits. Comparison of frequencies will be performed by Pearson chi-square test. Comparison of differences between mean values will be performed with Student’s t-test or Mann–Whitney test. Correlations between the main continuous variables will be assessed using the parametric Pearson or the nonparametric Spearman’s Rho correlation coefficient. Linear regression and logistic regression analysis will be performed to investigate whether treatment with JAK inhibitors is associated with NVC parameters and parameters obtained from 24h ambulatory blood pressure recording after adjustment for other variables. A probability value of p≤0.05 will be considered statistically significant.

## ANTICIPATED BENEFITS

Despite their clinical efficacy in alleviating symptoms and progression of RA, cardiometabolic effects of JAK inhibitors in patients with RA remain in comparison underinvestigated, especially in the long-term. JAK inhibitors may confer increased risk for circulatory disorders, specifically thromboembolic events, and adversely affect lipids and platelet count.^19^ More recent evidence shows an increased risk of major adverse cardiovascular events associated with JAK inhibitors in RA, although the underlying mechanisms remain unclear.^17^ It has been argued that the patients’ aggravated cardiovascular profile with presence of multiple CVD risk factors may account, at least partially, for this observation.^18^ Hypertension is highly prevalent in RA and contributes substantially to the increased CVD burden associated with the disease,^11^ while ambulatory blood pressure monitoring is superior to conventional blood pressure measurements in terms of CVD risk prediction.^21^ Furthermore, subclinical microvascular injury is present in patients of RA before the establishment of overt CVD^3,4,51^ and is considered as an intermediate pathophysiological process towards the development of cardiovascular complications.^6^ At the same time, large artery stiffening represents a surrogate marker of CVD with incremental predictive value beyond traditional cardiovascular risk factors.^28^ However, the impact of JAK inhibition on blood pressure including its 24h variation, and markers of micro- and macrovascular injury has not been investigated so far in patients with RA.

Therefore, shedding light on the impact of JAK inhibitors on the above critical parameters (24h BP and day-to night-time variation, arterial stiffness, microangiopathy of the dermal capillary network) and the prothrombotic markers that will be assessed, will add substantial information to the available literature regarding cardiovascular aspects of JAK inhibitors in RA. It is anticipated that our findings will have direct clinical implications. They will either highlight potential pleiotropic beneficial effects of JAK inhibitors on the cardiovascular system, or by contrast detect and highlight potential negative aspects which should be taken into consideration by treating physicians, in order to take actions targeting at their proper monitoring and management.

Besides, evaluation of changes in patients’ haemodynamic profile and circulating biomarkers of thrombotic predisposition will point towards underlying pathophysiological mechanisms mediating cardiovascular effects of JAK inhibitors in RA. Considering the innovative study design, our results will most probably have an international impact following their dissemination in conferences and the press and their publication in peer-reviewed journals.
